# Finite-Element Modeling of the Temperature Effect on Extended Avalanche Damage of Gas Main Pipelines

**DOI:** 10.3390/ma17091963

**Published:** 2024-04-24

**Authors:** Nurlan Zhangabay, Ulzhan Ibraimova, Alpysbay Ainabekov, Svetlana Buganova, Arman Moldagaliev

**Affiliations:** 1 Department of Architecture and Urban Planning, M. Auezov South Kazakhstan University, Av. Tauke Khan, No. 5, Shymkent 160012, Kazakhstan; 2 Department of Industrial Civil and Road Construction, M. Auezov South Kazakhstan University, Av. Tauke Khan, No. 5, Shymkent 160012, Kazakhstan; ibraimova_uljan@mail.ru; 3 Scientific Research Laboratory “Problems of Mechanical Engineering”, M. Auezov South Kazakhstan University, Av. Tauke Khan, No. 5, Shymkent 160012, Kazakhstan; a.ainabekov52@rambler.ru; 4 Department of Building Technologies, Infrastructure and Management International Education Corporation (KazGASA), Ryskulbekov Str., 28, Almaty 050043, Kazakhstan; svetlanabuganova@gmail.com; 5 Department of Mechanics and Mechanical Engineering, M. Auezov South Kazakhstan University, Av. Tauke Khan, No. 5, Shymkent 160012, Kazakhstan; arm_mold81@mail.ru

**Keywords:** steel pipeline, straight-through crack, crack-like defect, thermal properties, thermo-elasticity, finite-element model

## Abstract

The dynamic stress–strain state and fracture of a steel main gas pipe section between supports with a straight-through crack was analyzed with consideration of the temperature effect on changes in the mechanical properties of the pipe material. The numerical solution of the problem was implemented in the ANSYS-19.2/Explicit Dynamics software package. The process of fracture in a section of the gas pipeline “Beineu–Bozoy–Shymkent” with a linear crack in the temperature range of −40 °C to +50 °C at the operating pressure of 7.5 MPa and critical pressure equal to 9.8 MPa was considered. As a result, it was found that at the initial growth of the internal pressure from working pressure to critical pressure, the length of the crack doubled. At the same time, the process had a local characteristic. Further development of the crack had the nature of avalanche fracture and depended on the temperature of the steel pipeline. With increasing temperature, there was also an increase in the length of the crack at the avalanche fracture. Thus, at a temperature of 40 °C, the crack lengthened 67.75-fold; at a temperature of −10 °C, the crack lengthened 68-fold; at a temperature of +20 °C, the crack lengthened 68.25-fold; and at a temperature of +50 °C, the crack lengthened 68.5-fold. In this work, this difference was 75% of the initial crack length. This fact will be used for further development of the technique of strengthening damaged pipe sections using bandages.

## 1. Introduction

During the operation of steel main gas pipelines, especially in seismically hazardous areas, failures are common. Failures, as a rule, arise under the influence of two groups of factors: a reduction in the bearing capacity of the pipeline under the influence of local defects, which are stress concentrators, and a pressure increase due to unsteady operating modes. The influence of various factors on the serviceability of main gas pipelines is uneven [1]. Avalanche failure is especially dangerous for pipes [2]. This can be caused by a number of reasons, such as mechanical impacts, emergency situations, natural phenomena (snowfalls, landslides, and earthquakes), and other factors that lead to the formation of cracks [3].

When describing the mechanism of gas pipeline fracture, three stages are distinguished: (1) crack nucleation and its stable state, (2) crack development up to the critical dimensions, and (3) propagation of a rapidly developing crack [4]. Prolonged avalanche fracture is the final stage of the gas main fracture process. Cracks along gas pipelines can have different shapes depending on the type of material, the internal and external loads, the causes of occurrence, and other factors [5]. The main classical forms of cracks along the main gas pipelines include straight cracks, branching cracks, cross cracks, radial cracks, collapsing cracks, fatigue cracks, and corrosion cracks [6]. Straight cracks have a straight shape and can propagate along the pipeline without deviation. They are characterized by the fact that their walls are identical and parallel to each other. Straight cracks are the most dangerous from the point of view of the extended avalanche fracture of main gas pipelines.

The temperature plays a significant role in the process of main gas pipeline fracture. The reasons for this influence are thermal stresses in the structure and changes in the mechanical properties of the pipe material at different temperatures. Temperature changes cause the expansion and contraction of the materials that make up the pipeline. This contributes to thermal stresses in the material, especially in the presence of defects. When the temperature changes, properties such as the strength, elasticity, and plasticity change [7,8]. This, in turn, affects the ability of the pipeline to resist mechanical loads and deformations, changing the nature of the fracture processes. In general, understanding the influence of temperature on the strength of the structure is important in the development of measures to ensure the safety and reliability of the operation of gas pipelines with crack-like defects.

Numerous scientists have dealt with the problem of the strength of gas pipelines, which is due to its great practical relevance. Marvin [9] described the results of experimental studies on fatigue crack development in the areas of residual stresses of rupture and compression. In the review article presented by Nnoka et al. [10], approaches accounting for the influence of residual stresses by analytical methods were considered. Chen et al. [11] evaluated the mechanical behavior of a damaged steel pipeline using finite-element analysis. James and Hudgins [2], Zhangabay et al. [5], Olamide et al. [12], and Stepova O. et al. [13] analyzed the prediction and prevention of external corrosion. Different forms of external corrosion and failure mechanisms were considered. The study presented by Hutař et al. [14], based on linear elastic fracture mechanics methods, proposed a methodology for the numerical estimation of the service life of polymer pipes under internal pressure. The study proposed by Elyasi et al. [15] investigated the ductile fracture properties of API 5L and X52 pipeline steels using the finite-element method in ABAQUS. The work developed by Arumugam et al. [16] presented an overview of the component failures of corroded steel pipes subjected to internal pressure and axial compressive stress. The study presented by Kantor et al. [17] investigated the propensity of X80 low-carbon micro-alloyed pipeline steels to brittle fracture at low temperatures. The experimental results of impact toughness variation, based on multiple Charpy fracture tests in the temperature range from 20 °C to −1000 °C, were presented. It was experimentally established that the reason for the scatter of impact toughness values during the fully plastic fracture of specimens, as well as during fracture accompanied by the formation of separations, was the local heterogeneity of plastic properties. The greater the propensity to form separations for a particular steel, the lower the impact toughness. In Martynenko et al. [18], the effect of temperature ranging from −40 °C to +50 °C on the strength of the steel elements of a composite structure was shown.

The internal pressure along the main pipeline varies in value and is one of the main loads causing fatigue of the material. It is one of the main causes of accidents and destruction in the linear part of the main pipeline. The operating pressure causes tangential and normal stresses along the pipe walls, which can change when the internal pressure of the working medium increases or decreases. Under the action of test pressure along pipelines, stresses arise, due to which the probability of pipe failure with defects reaches a high level. Sarker and Sarker [19] demonstrated an approach to estimate the power of the pressure wave generated by hydrostroke. In addition, Nordhagen et al. [20] presented an experimentally obtained time of action and pressure decay rate in a cracked pipe with methane at a pressure equal to 12.2 MPa.

According to the aforementioned literature review, the following conclusions can be drawn. The task of modeling the influence of temperature on the process of extended avalanche fracture in large-diameter gas pipelines with rectilinear cracks is urgent. To date, the issue of crack propagation along the sections between supports at different temperatures remains poorly studied and requires further research. Understanding the influence of temperature on the propagation range of straight cracks during avalanche fracture will make it possible to properly organize measures for strengthening pipeline sections with defects. In this regard, at this stage of the present work, the main objective of this contribution was to analyze the dynamic stress–strain state and fracture of pipe sections between supports with a linear crack, taking into account the effect of temperature on changes in the mechanical properties of the pipe material.

## 2. Materials and Methods

### 2.1. Mathematical Model of Dynamic Stress–Strain State and Fracture of Steel Pipelines with Consideration of Temperature Influence

**Figure 1:**
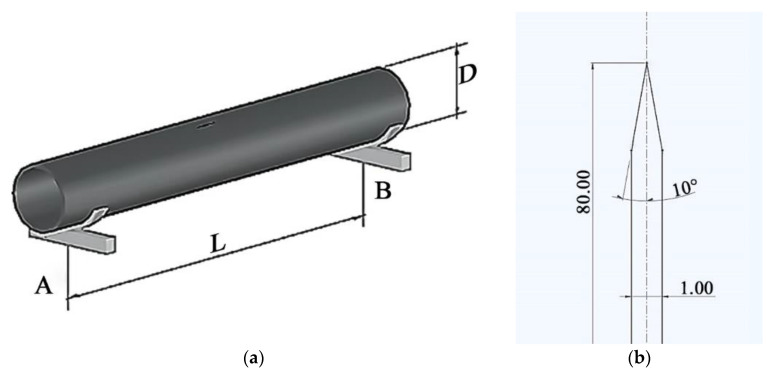
Diagram of a gas pipeline section with a crack between supports: (**a**) overall view and (**b**) top of the crack.

A section of length L of the main gas pipeline between two supports was considered (Figure 1a). A pipe with a large diameter D and wall thickness H was assumed. There was a straight crack in the central part of the pipe on its outer surface. It ran in the longitudinal direction of the pipe, perpendicular to the axial section. Its initial width w did not exceed a value of 1.0 mm or an initial length of *l* = 80 mm. A variant of the crack a = H was also considered, where the angle at the crack tip at the initial moment of the dynamic process was instead equal to 20° (Figure 1b). One support implemented the conditions of rigid pipe embedment, while the other moved longitudinally. The supports were equidistant from the initial position of the crack for a long distance and did not influence the process of its development.

The variable load of the considered gas pipeline section was modeled. The internal pressure drop in the pipe during the formation of a through crack caused by gas leakage and the gas pressure on the walls of the through crack were considered.

The pipe inner surface was loaded with uniformly distributed dynamic pressure $P\left(t\right)$ [20]. It was considered that the beginning of the dynamic process *t*_0_ was the moment when the fixed operating pressure along the gas pipe began to change. The value of the dynamic pressure at any time was defined as follows [20]:

(1) $$P\left(t\right)=P_{max}e^{-H\left(t-t_{max}\right)\cdot t/\theta },$$

where $P_{max}$ is the pressure in the pipe operating until the formation of a through crack, $t_{max}$ is the moment of time for the formation of a through crack, $H\left(t-t_{max}\right)$ is the Heaviside function, and θ is the gas pressure attenuation coefficient along the pipe.

Gas-dynamic processes taking place in the vicinity of a through crack in a gas pipeline are extremely complex [20]. A number of models of gas flow through a crack currently exist. The model that most accurately describes the process in this work is the one that considers fracture deformation because the crack can change its shape and size as the pressure changes during gas flow through a crack [21]. At the same time, progressive uniform gas flow is disturbed in the area of the crack, and as a result, the pressure distribution in the pipe cross-section of the damage zone is not uniform. However, the major difficulty in modeling gas pressure changes in the damage zone is the impossibility of mathematically describing the processes of gas flow into the atmosphere through a formed crack due to the uncertainty of its size and topography. Consequently, it is appropriate to use a coefficient value of θ based on experimental studies [20].

The temperature effect on the dynamic deformation and local damage of the steel gas pipeline section was considered. The temperature had a major role in the process of destroying the main gas pipeline. Thus, the main effects included the following:

- Thermal stresses: Changes in the temperature cause the expansion and contraction of the materials that form the pipeline. This can generate stresses in the material, especially when it contains defects. Long-term thermal stresses can lead to the development of cracks and other damage.
- Changes in the material’s mechanical properties: When the temperature changes, the physical properties of the materials, such as their strength, elasticity, and plasticity, change. This can influence the ability of the pipeline to resist mechanical loads and deformations.
- Cryogenic effects: Sometimes, gases, such as liquefied natural gas, are transported at very low temperatures. This may cause cryogenic effects, such as ice formation and condensation, which can affect pipelines’ integrity.
- Corrosion: High or low temperatures can increase the corrosion process of the pipeline material. For instance, wet conditions at low temperatures can promote ice formation and accelerate the corrosion process.

The process of the avalanche damage in cracked sections of gas pipelines is a fast-moving process [21]. Therefore, the long-term temperature effect in this problem can be disregarded, and only the temperature effect on the mechanical properties of the pipe material can be considered [22]. This effect was assumed in the mathematical model of the task. Specifically, the material mechanical constants were considered as functions of the temperature: Young’s modulus *E* = *E*(*T*), ultimate stress limit $\sigma_{B}$ = $\sigma_{B}\left(T\right)$, and yield strength $\sigma_{T}$ = $\sigma_{T}\;\left(T\right)$, where T is the pipe material temperature.

Movements, plastic deformations, and equivalent stresses as functions of time were determined. The material crack growth over time and local damage in the crack tip zone were analyzed. An analysis of the stress–strain state and local damage of a steel pipe section with a crack was carried out using 3D modeling.

The equations of the dynamics, excluding mass forces in a cylindrical coordinate system (*r*, *φ*, *z*), take the following forms [23,24]:

$$\frac{\partial \sigma_{r}}{\partial r}+\frac{\sigma_{r}-\sigma_{\phi }}{r}+\frac{\partial \tau_{r\phi }}{r\partial \phi }+\frac{\partial \tau_{rz}}{\partial z}=\rho \frac{\partial^{2}u}{\partial t^{2}},$$

(2) $$\frac{\partial \sigma_{\phi }}{r\partial \phi }+\frac{\partial \tau_{r\phi }}{\partial r}+\frac{2\partial \tau_{r\phi }}{r}+\frac{\partial \tau_{\phi z}}{\partial z}=\rho \frac{\partial^{2}v}{\partial t^{2}},$$

$$\frac{\partial \sigma_{z}}{\partial z}+\frac{\partial \tau_{rz}}{\partial r}+\frac{\tau_{rz}}{r}+\frac{\partial \tau_{z\phi }}{r\partial \phi }=\rho \frac{\partial^{2}w}{\partial t^{2}},$$

where $\sigma_{r}$, $\sigma_{\phi }$, and $\sigma_{z}$ are the components of normal stress; $\tau_{r\phi }$, $\tau_{rz}$,$\;\mathrm{and}\;\tau_{z\phi }$ are the components of shear stress; u, v, and w are the movement components; and ρ is the material density. The boundary conditions were equal, as follows: $\sigma_{r_{r=D/2}=}-P\left(t\right),\;\sigma_{\Sigma }=-P\left(t\right)$, where Σ is the surface of the crack edges. All remaining boundary conditions and initial conditions were instead assumed to be equal to zero. Particularly, the stress components in Equation (2) were determined depending on the deformation stage:

$$\sigma_{r}-\sigma_{0}=\frac{1}{\psi }\left(\varepsilon_{r}-\frac{1}{3}\varepsilon_{0}\right),\;\tau_{r\phi }=\frac{1}{\psi }\gamma_{r\phi },$$

(3) $$\sigma_{\phi }-\sigma_{0}=\frac{1}{\psi }\left(\varepsilon_{\phi }-\frac{1}{3}\varepsilon_{0}\right),\;\tau_{\phi z}=\frac{1}{\psi }\gamma_{\phi z},$$

$$\sigma_{z}-\sigma_{0}=\frac{1}{\psi }\left(\varepsilon_{z}-\frac{1}{3}\varepsilon_{0}\right),\;\tau_{zr}=\frac{1}{\psi }\gamma_{zr},$$

$$\sigma_{0}=\frac{1}{3}\left(\sigma_{r}+\sigma_{\phi }+\sigma_{z}\right),$$

$$\varepsilon_{0}=\varepsilon_{r}+\varepsilon_{\varphi }+\varepsilon_{z}.$$

The problem was solved with the elastic–plastic formulation. In the elastic deformation case, $\psi =\frac{1}{2\mu }$, and the dependences (3) correspond to Hooke’s law. The equation of the state under elastic deformation of a material with regard to the temperature effect is reported as follows: $\sigma_{eq}=E\left(T\right)\cdot \varepsilon_{eq}$, where $E\left(T\right)$ is Young’s modulus for a specified temperature, $\sigma_{eq}$ is the equivalent stress, and $\varepsilon_{eq}$ is the equivalent deformation. In the plastic deformation case, $\psi =\frac{3}{2}\frac{\varepsilon_{eq}}{\sigma_{eq}\left(\varepsilon_{eq}\right)}$. The equation of the state under plastic deformation, with the deformation rate not exceeding 10^−2^ c^−1^, was instead modeled using the dependence of bilinear isotropic hardening (BIH) [25]. In this case, an equation of the state with regard to the temperature effect was as follows: $\sigma_{eq}=\sigma_{y}\left(T\right)+H\left(\varepsilon_{eq}-\sigma_{y}\left(T\right)/E\left(T\right)\right)$, where $\sigma_{y}\left(T\right)$ is the yield strength for a specified temperature and $H=d\sigma_{eq}/d\varepsilon_{eq}$ is the hardening modulus. In the speedy plastic deformation case, $\psi =\frac{3}{2}\frac{\varepsilon_{eq}}{\sigma_{eq}\left(\varepsilon_{eq},{\dot{\varepsilon }}_{eq}\right)}$, where ${\dot{\varepsilon }}_{eq}$ is the deformation rate. When the deformation rate exceeds the value of 10^−2^ c^−1^, the plastic flow of the material is described using the Cooper–Symonds model (CSS) [25]. The use of this model allows us to take into consideration the effect of the deformation rate on the plastic flow process of the material. The equation of the state is reported as follows: $\sigma_{eq}=\left[A\left(T\right)+B\left(T\right)\cdot {\left(\varepsilon_{eq}^{pl}\right)}^{n}\right]\cdot \left[1+{\left(D^{-1}\cdot \partial \varepsilon_{eq}^{pl}/\partial t\right)}^{1/q}\right]$, where $A\left(T\right)$ is the yield strength at zero plastic deformation for a specified temperature; *B*$\left(T\right)$ is the hardening coefficient; $\varepsilon_{eq}^{pl}$ is the plastic deformation; *n* is the hardening index; $\partial \varepsilon_{eq}^{pl}/\partial t$ is the plastic deformation rate; and *D*, *q* are the hardening coefficients at the deformation rate. The dependencies between the deformations and displacements in a cylindrical coordinate system (*r*, *φ*, *z*) are nonlinear [23,24]:

$$\varepsilon_{r}=\frac{\partial u}{\partial r}+\frac{1}{2}\left({\left(\frac{\partial u}{\partial r}\right)}^{2}+{\left(\frac{\partial v}{\partial r}-\frac{v}{r}\right)}^{2}+{\left(\frac{\partial w}{\partial r}\right)}^{2}\right),$$

$$\varepsilon_{\phi }=\frac{\partial v}{r\partial \phi }+\frac{u}{r}+\frac{1}{2}\left({\left(\frac{\partial v}{r\partial \phi }+\frac{u}{r}\right)}^{2}+{\left(\frac{\partial u}{r\partial \phi }\right)}^{2}+{\left(\frac{\partial w}{r\partial \phi }\right)}^{2}\right),$$

(4) $$\varepsilon_{z}=\frac{\partial w}{\partial z}+\frac{1}{2}\left({\left(\frac{\partial u}{\partial z}\right)}^{2}+{\left(\frac{\partial v}{\partial z}\right)}^{2}+{\left(\frac{\partial w}{\partial z}\right)}^{2}\right),$$

$$\gamma_{r\phi }=\frac{\partial u}{r\partial \phi }+\frac{\partial v}{\partial r}-\frac{v}{r}+\frac{1}{2}\left(\frac{\partial u}{r\partial \phi }\frac{\partial u}{\partial r}+\left(\frac{\partial v}{r\partial \phi }+\frac{u}{r}\right)\left(\frac{\partial v}{\partial r}-\frac{v}{r}\right)+\frac{\partial w}{r\partial \phi }\frac{\partial w}{\partial r}\right),$$

$$\gamma_{\phi z}=\frac{\partial v}{\partial z}+\frac{\partial w}{r\partial \phi }+\frac{1}{2}\left(\frac{\partial v}{\partial z}\left(\frac{\partial v}{r\partial \phi }+\frac{u}{r}\right)+\left(\frac{\partial u}{\partial z}\frac{\partial u}{r\partial \phi }\right)+\frac{\partial w}{\partial z}\frac{\partial w}{r\partial \phi }\right),$$

$$\gamma_{rz}=\frac{\partial u}{\partial z}+\frac{\partial w}{\partial r}+\frac{1}{2}\left(\frac{\partial u}{\partial z}\frac{\partial u}{\partial r}+\frac{\partial u}{\partial z}\left(\frac{\partial v}{\partial r}-\frac{v}{r}\right)+\frac{\partial w}{\partial z}\frac{\partial w}{\partial r}\right),$$

where ε*_r_*, ε*_φ_*, ε*_z_* are the normal components of the deformation tensor, and γ*_rφ_*, γ*_φz_*, γ*_rz_* are the tangent components of the deformation tensor. The equivalent deformations were conversely obtained by the components of the deformation tensor (4) [21], which are described as follows:

(5) $$\varepsilon_{eq}=\frac{2}{3}\sqrt{\frac{3\left(e_{r}^{2}+e_{\phi }^{2}+e_{z}^{2}\right)}{2}+\frac{3\left(\gamma_{r\phi }^{2}+\gamma_{\phi z}^{2}+\gamma_{zr}^{2}\right)}{4}},$$

$$e_{rr}=+\frac{2}{3}\varepsilon_{r}-\frac{1}{3}\varepsilon_{\phi }-\frac{1}{3}\varepsilon_{z}$$

$$e_{22}=-\frac{1}{3}\varepsilon_{r}+\frac{2}{3}\varepsilon_{\phi }-\frac{1}{3}\varepsilon_{z}$$

$$e_{33}=-\frac{1}{3}\varepsilon_{r}-\frac{1}{3}\varepsilon_{\phi }+\frac{2}{3}\varepsilon_{z}$$

The material damage was modeled based on a maximum stress criterion (von Mises criterion) [26]. When the equivalent stresses exceed the limit stresses, $\sigma_{eq}>\sigma_{y}\left(T\right)$, local destruction of the structural material occurs [27].

### 2.2. Finite-Element Model of Dynamic Stress–Strain State and Damage of a Steel Pipe Section with Regard to the Temperature Effect on the Mechanical Characteristics of the Material

A numerical solution of the problem was performed in the software package ANSYS-19.2/Explicit Dynamics. Here, spatial discretization was based on the finite-element method according to the equation of movement in the form of

(6) $$\left[M\right]\left\{\ddot{u}\right\}+\left[K\left(\varepsilon_{i},{\dot{\varepsilon }}_{i}\right)\right]\left\{u\right\}=\left\{F\right\}.$$

where $\left[M\right]$ is the mass matrix of the finite-element model, $\left\{u\right\}$ is the vector of the generalized nodal movements of the finite-element model, $\left[K\right]=\left[K\left(T\right)\right]$ is the stiffness matrix of the finite-element model for a specified temperature, and $\left\{F\right\}$ is the vector of forces compressed to the nodes.

The central second-order differential integration scheme was the basis for time discretization, based on which the values of accelerations, velocities, and movements were calculated as follows [25]:

$${\left\{\ddot{u}\right\}}_{n}={\left[M\right]}^{-1}\left(\left\{F\right\}-\left[K\left(\varepsilon_{i}\left(\left\{u_{n}\right\}\right),{\dot{\varepsilon }}_{i}\left(\left\{u_{n}\right\}\right)\right)\right]\left\{u_{n}\right\}\right),$$

(7) $${\left\{\dot{u}\right\}}_{n+\frac{1}{2}}={\left\{\dot{u}\right\}}_{n-\frac{1}{2}}+{\left\{\ddot{u}\right\}}_{n}\Delta t,$$

$${\left\{u\right\}}_{n+1}={\left\{u\right\}}_{n}+{\left\{\dot{u}\right\}}_{n+\frac{1}{2}}\Delta t.$$

This system is steady in cases where the time integration step does not exceed $\Delta t_{cr}=\frac{2}{\omega_{max}}$, in which $\omega_{max}$ is the maximum natural frequency of the system $\omega {\frac{2c}{\Delta x_{min}}}_{max}$, *c* is the sound speed in the material; $\Delta x_{min}$ is the minimum typical size of the elements. A description of the movement of a deformed continuous environment was based on a multicomponent Lagrangian–Eulerian approach, which describes the flow of material through a grid moving in space [25]. A one-component Lagrangian–Eulerian approach and Lagrangian formulation were also adopted. The mass matrix $\left[M\right]$ in Equation (6) was achieved from an expression of the deformation of the structural elements for kinetic energy, $T=\frac{1}{2}{\int \int \int }_{V}\rho \left({\dot{u}}^{2}+{\dot{v}}^{2}+{\dot{w}}^{2}\right)dxdydz$, where *V* is the body volume and ρ is the material density. At finite-element discretization, the kinetic energy of deformation is reported as follows:

(8) $$T=\frac{1}{2}{\int \int \int }_{V}\rho {\dot{\upsilon }}^{T}N^{T}N\dot{\upsilon }dV=\frac{1}{2}{\dot{\upsilon }}^{T}M_{e}\dot{\upsilon },$$

where u=υN is the movement, υ is the vector of the nodal movement components, and *N* is the shape function matrix determining the position of the node elements. Specifically, the matrix of the element masses is equal to

(9) $$M_{e}={\int \int \int }_{V_{e}}\rho N_{e}^{T}N_{e}dV.$$

The stiffness matrix $\left[K\right]$ was obtained from the expression for the internal virtual work:

(10) $$\delta W=\int_{V}\sigma_{ij}\delta \varepsilon_{ij}dV,$$

where *W* is the internal virtual work. For a nonlinear system, potential energy accumulates over time and is usually not clearly expressed as a potential function of shift or velocity.

The Lagrange method was instead utilized to model the geometric nonlinearities. The problem was solved for a set of linearized synchronous equations with movement as the initial data were unknown for obtaining the solution at the moment of time *t* + Δ*t*. These synchronous equations were determined from an expression for the elements according to the principle of the virtual operation:

(11) $$\int_{V}\sigma_{ij}\delta \varepsilon_{ij}dV=\int_{V}f_{i}^{B}\delta u_{i}dV+\int_{S}f_{i}^{S}\delta u_{i}dS,$$

where *σ_ij_* is the Cauchy stress tensor component, $\varepsilon_{ij}=\frac{1}{2}\left(\frac{\partial u_{i}}{\partial x_{j}}+\frac{\partial u_{j}}{\partial x_{i}}\right)$ is the deformation tensor, *u_i_* is the deformation tensor, *x_i_* is the current coordinate, $f_{i}^{B}$ is the component of volumetric force, $f_{i}^{S}$ is the component of surface forces, *V* is the volume of the deformed body, and *S* is the surface of a deformed body on which the load acts. The expressions for the elements were achieved with differentiation of Equation (11). The linear differential terms were kept, and all higher-order terms were neglected. Thus, a linear system of equations was obtained.

In the finite-element setting, the constitutive physical relations were used to generate a relationship between the stress increment and the deformation increment. The law only reflects the increment of stresses through strains. However, Cauchy stresses depend on the rotation of a solid body and are not invariant. Hence, an expression was used to apply the necessary stresses, such as the Yaumann velocity of Cauchy stresses, to determine the physical relations ${\dot{\sigma }}_{ij}^{J}={\dot{\sigma }}_{ij}-\sigma_{ik}{\dot{\omega }}_{jk}-\sigma_{jk}{\dot{\omega }}_{ik}$, where ${\dot{\sigma }}_{ij}^{J}$ is the Yaumann velocity of Cauchy stresses, ${\dot{\omega }}_{ij}=\frac{1}{2}\left(\frac{\partial \upsilon_{i}}{\partial x_{j}}-\frac{\partial \upsilon_{j}}{\partial x_{i}}\right)$ is the rotation tensor, and ${\dot{\sigma }}_{ij}$ is the time derivative of Cauchy stresses. Therefore, the Cauchy stress velocity is described as follows:

(12) $${\dot{\sigma }}_{ij}={\dot{\sigma }}_{ij}^{J}+\sigma_{ik}{\dot{\omega }}_{jk}+\sigma_{jk}{\dot{\omega }}_{ik}$$

Using the basic physical relations, the stress change through deformation can be expressed as

(13) $${\dot{\sigma }}_{ij}^{J}=c_{ijkl}{\dot{\varepsilon }}_{kl},$$

where $c_{ijkl}$ is the tensor of the material constants, ${\dot{\varepsilon }}_{ij}=\frac{1}{2}\left(\frac{\partial v_{i}}{\partial x_{j}}+\frac{\partial v_{j}}{\partial x_{i}}\right)$ is the deformation velocity tensor, and $v_{i}$ is the velocity. The Cauchy stress velocity can instead be written as

(14) $${\dot{\sigma }}_{ij}=c_{ijkl}{\dot{\varepsilon }}_{kl}+\sigma_{ik}{\dot{\omega }}_{jk}+\sigma_{jk}{\dot{\omega }}_{ik}$$

The classical setting of net movements only considers the movement or velocity as the primary unknown variables. All the other quantities, such as deformations, stresses, and state variables in the history-dependent models of materials, were obtained from the movements. This formulation is the most widely used and can cope with the most nonlinear deformation tasks. Differentiation of δW allowed us to obtain the following expression:

(15) $$D\delta W=\int_{V}\left(D\sigma_{ij}\delta \varepsilon_{ij}dV+\sigma_{ij}D\delta \varepsilon_{ij}dV+\sigma_{ij}\delta \varepsilon_{ij}D\left(dV\right)\right)$$

Equation (9) was obtained as follows:

(16) $$D\sigma_{ij}=c_{ijkl}D\varepsilon_{kl}+\sigma_{ik}D\omega_{jk}+\sigma_{jk}D\omega_{ik},$$

where $D\omega_{ij}=\frac{1}{2}D\left(\frac{\partial u_{i}}{\partial x_{j}}-\frac{\partial u_{j}}{\partial x_{i}}\right)$. Differentiation was applied as follows: $D\left(dV\right)=\frac{\partial Du_{k}}{\partial x_{k}}dV=De_{v}dV$, where *e_v_* = *e_ii_*. By substituting Equations (11) and (12) into Equation (10), the following was obtained:

$$D\delta W=\int_{V}\delta \varepsilon_{ij}c_{ijkl}D\varepsilon_{kl}dV+\int_{V}\sigma_{ij}\left(\frac{\partial \delta u_{k}}{\partial x_{i}}\frac{\partial Du_{k}}{\partial x_{j}}-2\delta \varepsilon_{ik}D\varepsilon_{kj}\right)dV+\int_{V}\delta \varepsilon_{ij}\sigma_{ij}\frac{\partial Du_{k}}{\partial x_{k}}dV.$$

The third term is asymmetric and usually insignificant in most deformation cases. Hence, it was neglected. Consequently, the final formulation of the net movement is described as follows:

(17) $$D\delta W=\int_{V}\delta \varepsilon_{ij}c_{ijkl}D\varepsilon_{kl}dV+\int_{V}\sigma_{ij}\left(\frac{\partial \delta u_{k}}{\partial x_{i}}\frac{\partial Du_{k}}{\partial x_{j}}-2\delta \varepsilon_{ik}D\varepsilon_{kj}\right)dV.$$

The aforementioned Equation (17) is a set of linear equations with additive *Du_i_* or variable movements that can be solved using the standard linear solutions of McMeekin and Rice [25]. For the finite-element implementation of the general dynamic deformation model, its peculiarities should be accounted for. The limit conditions in element nodes must meet the equality of movements and their derivatives. In this case, the functions of shapes make it possible to describe a continuous and smooth change in stresses. The computational module Explicit Dynamics uses quadrilateral elements (tetrahedrons) and octahedral elements (hexahedrons) for 3D models. In this work, the hexahedral 8-node element was used, which provided equality of the movements, velocities, and accelerations at the nodes [25]. The convergence of the calculated finite-element model of the task was verified using a standard mesh densification method [21,25].

### 2.3. Initial Data for Finite-Element Modeling

**Figure 2:**
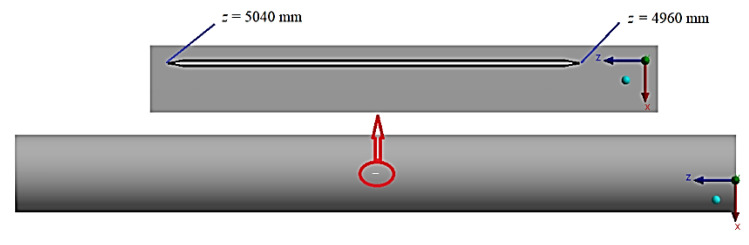
Diagram of crack location.

Finite-element numerical studies of the temperature effect on prolonged avalanche damage of main gas pipelines were carried out on an example of a section between the supports of the main gas pipeline “Beineu–Bozoy–Shymkent”. The distance between the supports was 36 m, while a rectilinear pipe section with a length of *L* = 10 m, a pipe inner diameter of *D* = 1.047 m, and a pipe wall thickness of *H* = 15.9 mm was assumed. There was a rectilinear crack in the central part of the studied section (Figure 2). Its initial width was *w* = 1.0 mm, while its initial length was *l* = 80 mm. A through crack with a depth of *a = H* was considered. An angle at the crack top at the initial moment of time was instead equal to 20°. When constructing a finite-element model, the convergence of the solution was checked. Based on the results of this check, a grid was generated with the value of the “Element Size” parameter equal to 0.01, which is described in detail in the previous work of the authors [21].

A section of pipe with a crack is under a load of unsteady internal pressure. The pressure variation with time was given in tabular form according to the experimental data obtained by Nordhagen et al. [20]. A process of internal pressure changes in the pipe from the operating to the critical pressure, and the subsequent pressure drop due to the gas flow through the crack, was modeled. Table 1 shows the estimated values of the internal pressure over time.

The pipe material was steel X70 [28]. The steel density was taken as ρ = 7810 kg/m^3^, and the Poisson’s ratio was μ= 0.3. The mechanical characteristics of steel, the Young’s modulus *E*, tensile strength $\sigma_{U},$ and yield strength $\sigma_{y}$, depend on the temperature. Studies were conducted within the temperature range of −40 °C to +50 °C. Table 2 shows the discrete values of these parameters for a specified temperature range with a step of 30 °C, which were adopted for the calculation studies. These values were gained by linear interpolation of the experimental data [18,29]. It is noted that X70 steel is a high-strength low-carbon micro-alloyed pipeline steel with high impact strength at low temperatures, while a temperature lower than −40 °C does not induce structural changes, leading to metal brittleness [18,29].

## 3. Results and Discussion

### 3.1. Study of a Rectilinear Section of a Gas Pipeline with a Crack at a Temperature of −40 °C

**Figure 3:**
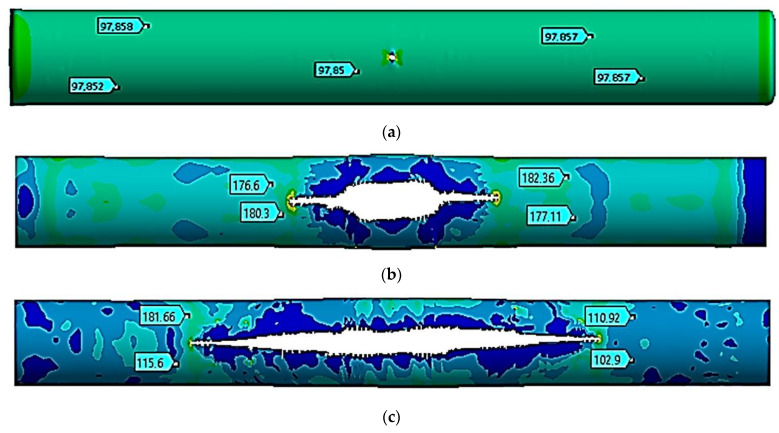
Equivalent stresses at a temperature of −40 °C (unit: MPa). (**a**)—*t* = 1.0 ms, (**b**)—*t* = 10 ms, and (**c**)—*t* = 20 ms.

The stress state of a pipe section with a crack was studied at a temperature of −40 °C. Figure 3 shows the movements in the pipe at the following moments: the start of the critical pressure impact at *t =* 1 ms (Figure 3a), the maximum crack opening at *t =* 10 ms (Figure 3b), and the stop of the crack growth at *t =* 20 ms (Figure 3c).

Table 3 shows the values of the *z* coordinate for the left and right crack ends at the specified moments. The distance between the ends was determined.

### 3.2. Study of a Rectilinear Section of a Gas Pipeline with a Crack at a Temperature of −10 °C

**Figure 4:**
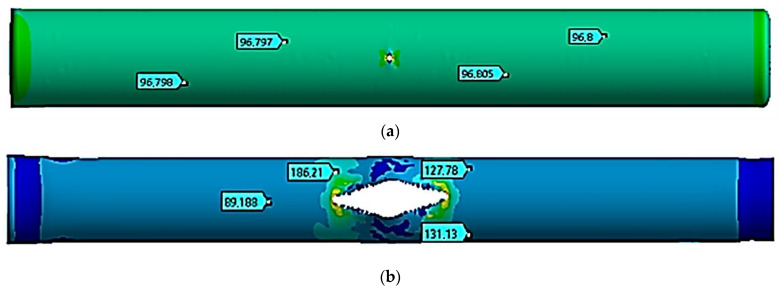

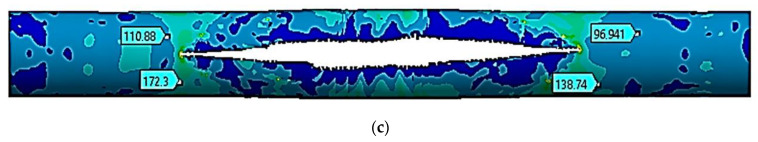
Equivalent stresses at a temperature of −10 °C (unit: MPa). (**a**)—*t* = 1.0 ms, (**b**)—*t* = 8.0 ms, and (**c**)—*t* = 20 ms.

The stress state of a pipe section with a crack was also studied at a temperature of −10 °C. Figure 4 shows the movements in the pipe at the following moments: the start of the critical pressure impact at *t =* 1.0 ms (Figure 4a), the maximum crack opening at *t =* 8.0 ms (Figure 4b), and the stop of the crack growth at *t =* 20 ms (Figure 4c).

Table 4 shows the values of the *z* coordinate for the left and right crack ends at the specified moments of time. The distance between the ends was obtained.

### 3.3. Study of a Rectilinear Section of a Gas Pipeline with a Crack at a Temperature of +20 °C

**Figure 5:**
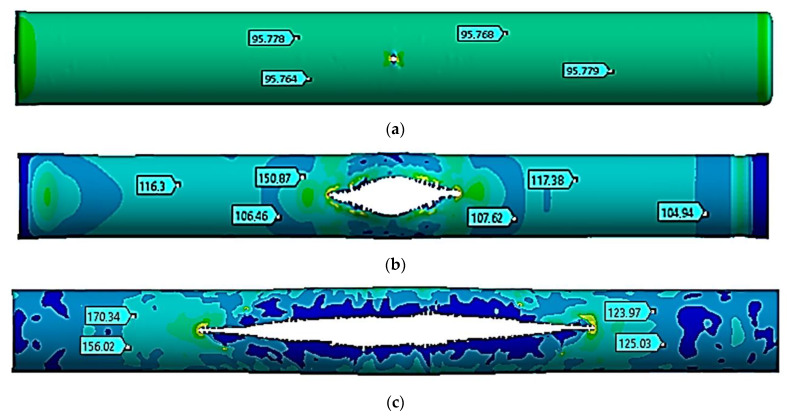
Equivalent stresses at a temperature of +20 °C (unit: MPa). (**a**)—*t* = 1.0 ms, (**b**)—*t* = 6.0 ms, and (**c**)—*t* = 20 ms.

The stress state of a pipe section with a crack was also studied at a temperature of +20 °C. Figure 5 shows the movements in the pipe at the following moments: the start of the critical pressure impact at *t =* 1.0 ms (Figure 5a), the maximum crack opening at *t =* 6.0 ms (Figure 5b), and the stop of the crack growth at *t =* 20 ms (Figure 5c).

Table 5 shows the values of the *z* coordinates for the left and right crack ends at the specified moments of time. The distance between the ends was similarly determined.

### 3.4. Study of a Rectilinear Section of a Gas Pipeline with a Crack at a Temperature of +50 °C

**Figure 6:**
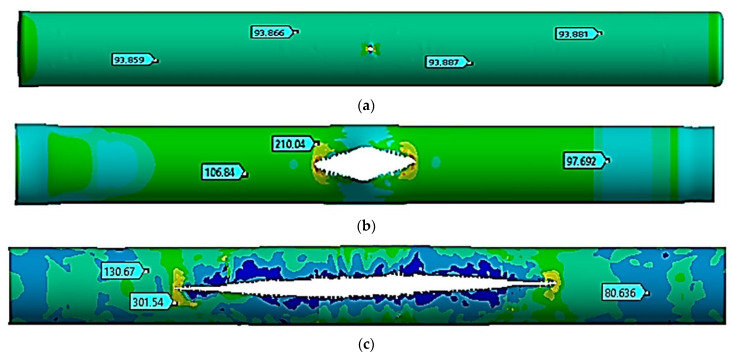
Equivalent stresses at a temperature of +50 °C (unit: MPa). (**a**)—*t* = 1.0 ms, (**b**)—*t* = 5.0 ms, and (**c**)—*t* = 20 ms.

The stress state of a pipe section with a crack was finally analyzed at a temperature of +50 °C. Figure 6 shows the movements in the pipe at the following moments: the start of the critical pressure impact at *t =* 1.0 ms (Figure 6a), the maximum crack opening at *t =* 5.0 ms (Figure 6b), and the stop of the crack growth at *t =* 20 ms (Figure 6c).

Table 6 shows the values of the *z* coordinate for the left and right crack ends at the specified moments of time. The distance between the ends was similarly determined.

The process of fracture of a gas pipeline section with a straight crack in the temperature range of −40 °C to +50 °C was considered. For all considered cases, the nature of the deformation and fracture had similar patterns. At the beginning of the process, there was a divergence in the crack edges, i.e., an increase in its width. This led to an increase in the crack opening angle at the crack tips and the stress concentration at the crack tips. Plastic deformations were observed at the crack tips, and stresses in this local zone exceeded the strength limit of the material. There was a local destruction of the material in the zones of the crack tips, which led to its growth. The process was symmetrical, which was explained by the symmetry of the geometrical model away from the fixed pipe ends. During the first millisecond, when the pressure increased from the working pressure of 7.5 MPa to the critical one equal to 9.8 MPa, the crack size doubled, from 80 mm to 160 mm. This was typical for the whole temperature range. At the same time, the stress state of the structure outside the crack zone was uniform, while the deformations were elastic.

The crack opening to the maximum crack width occurred differently for each temperature. Thus, at a temperature of −40 °C, the crack was maximally opened only by 10 ms. At the same time, the crack growth occurred in the longitudinal direction. At 10 ms, its length was already 2.84 m, which was 35.5 times that of the initial length. From 8.0 ms to 22 ms, the acting pressure was 2.5 MPa, which was 25.5% of the critical pressure. At the value of 20 ms, the crack growth stopped. Conversely, at a temperature of −40 °C, the crack elongated 67.75-fold and reached a size of 5.42 m. At temperatures equal to −10 °C, +20 °C, and +50 °C, the crack maximally opened within 8.0 ms, 6.0 ms, and 5.0 ms, respectively. For these three cases, the nature of the maximum crack opening was the same. At the beginning of the deformation process, the crack increased uniformly in both width and length. After the maximum opening, it started to increase rapidly along the longitudinal direction. Therefore, for a temperature of −10 °C within 8.0 ms, the crack elongated 34-fold. For a temperature of +20 °C within 6.0 ms, the crack elongated 23-fold, while for a temperature of +50 °C within 5.0 ms, the crack elongated 19-fold. Thus, for all the cases considered, crack growth ceased within a value of 20 ms. Furthermore, at a temperature of −10 °C, the crack elongated 68-fold and reached a size of 5.44 m. At a temperature of +20 °C, the crack elongated 68.25-fold and reached a size of 5.46 m. And at a temperature of +50 °C, the crack elongated 68.5-fold and reached a size of 5.48 m. According to these results, as the temperature increased, the crack length also increased. In this work, this difference was 60 mm, 75% of the initial length, and there was a temperature difference of 90 °C. As a limitation of this study, it can be noted that cracks in the gas pipeline were considered only in the straight section of the pipe. In the future, the authors plan to expand and continue research on the strength of sections of main gas pipelines with cracks reinforced with various types of bandages in order to prevent their avalanche destruction [5,8,30,31].

## 4. Conclusions

The dynamic stress–strain state and failure of a pipe section between supports with a straight-through crack were analyzed considering the effect of temperature on changes in the mechanical properties of the pipe material. The mathematical model of the problem took into account the three-dimensional geometry of the pipe with a through crack, internal pressure variations over time, crack deformation, plastic properties, and the effect of the temperature on the mechanical properties of the structural material. The corresponding finite-element solution of the problem was implemented within ANSYS-19.2/Explicit Dynamics software, and the spatial discretization was based on the finite-element method according to the equation of motion. Numerical simulations were carried out on an example of a section between the supports of the main gas pipeline “Beineu–Bozoy–Shymkent”. The pipe section with a straight-through crack was investigated. Particularly, investigations were executed for the temperature range from −40 °C to +50 °C. It was found that at the initial growth of the internal pressure from the working pressure to the critical point, the length of the through crack doubled. At the same time, the process had a local characteristic. This fact will be used for the development of a methodology for strengthening damaged pipe sections using bandages. Further development of the crack had the nature of avalanche fracture and depended on the temperature of the steel pipeline. Notably, with an increase in the temperature, there was also an increment in the crack length at avalanche fracture. At a temperature of −40 °C, the crack elongated 67.75-fold; at a temperature of −10 °C, the crack elongated 68-fold; at a temperature of +20 °C, the crack elongated 68.25-fold; and at a temperature of +50 °C, the crack elongated 68.5-fold. In this work, this difference was 75% of the initial crack length.

## Figures and Tables

**Table 1:** Values of the nonfixed internal pressure over time.

*t*, ms	0	1	2	3	4	5	6	7	8	22	30
*P(t)*, MPa	7.5	9.8	9.8	9.1	7.5	5.0	3.5	3.2	2.5	2.5	1.5

**Table 2:** Mechanical characteristics of steel within the temperature range of −40 °C to +50 °C.

Temperature, *T*, °C	−40	−10	+20	+50
Tensile strength, $\sigma_{U}$, MPa	574	572	570	568
Yield strength, $\sigma_{y}$, MPa	525	515	505	495
Modulus of elasticity, *E*, GPa	211	209	206	203.5

**Table 3:** Values of *z* coordinate for left and right crack ends at a temperature of −40 °C.

Moment in Time *t*, ms	0	1	10	20
Right end *z* coordinate, mm	4960	4920	3580	2290
Left end *z* coordinate, mm	5040	5080	6420	7710
Distance between ends, mm	80	160	2840	5420

**Table 4:** Values of *z* coordinate for left and right crack ends at a temperature of −10 °C.

Moment in Time *t*, ms	0	1	8	20
Right end *z* coordinate, mm	4960	4920	3640	2280
Left end *z* coordinate, mm	5040	5080	6360	7720
Distance between ends, mm	80	160	2720	5440

**Table 5:** Values of *z* coordinate for left and right crack ends at a temperature of +20 °C.

Moment in Time *t*, ms	0	1	6	19.5
Right end *z* coordinate, mm	4960	4920	4080	2270
Left end *z* coordinate, mm	5040	5080	5920	7730
Distance between ends, mm	80	160	1840	5460

**Table 6:** Values of *z* coordinate for left and right crack ends at a temperature of +50 °C.

Moment in Time *t*, ms	0	1	5	20
Right end *z* coordinate, mm	4960	4920	4240	2260
Left end *z* coordinate, mm	5040	5080	5760	7740
Distance between ends, mm	80	160	1520	5480

## Data Availability

Data are contained within the article.
